# Complete chloroplast genomes of endemic *Astragalus* and *Oxytropis* species from Uzbekistan

**DOI:** 10.12688/f1000research.178398.3

**Published:** 2026-06-01

**Authors:** Diyorjon Hamrayev, Bobur Karimov, Husniddin Esanov, Sodik Khuzhzhiev, Maxzuna Nasretdinova, Oysha Jabborova, Oybek Omonov, Mohistara Sharipova, Ziyoviddin Yusupov, Alijon Esankulov

**Affiliations:** 1 Institute of Botany, Academy of Sciences of Uzbekistan, Tashkent, Uzbekistan; 2 Bukhara State University, Bukhara, Uzbekistan; 3 Navoi State University, Navoi, Uzbekistan; 4 Samarkand State Medical University, Samarkand, Uzbekistan; 5 Bukhara state medical institute named after Abu Ali ibn Sino, Bukhara, Uzbekistan; 6 Karshi State University, Qarshi, Kashkadarya Province, Uzbekistan; 7 Turan University, Karshi, Uzbekistan; 8 Jizzakh State Pedagogical University, Jizzakh, Uzbekistan; 9 Tashkent Botanical Garden under the Institute of Botany, Academy of Sciences, Tashkent, Uzbekistan

**Keywords:** plastome; Fabaceae; IRLC; phylogeny; genome evolution; Central Asia

## Abstract

Chloroplast genomes provide important insights into plant phylogeny, genome evolution, and molecular marker development. In this study, we sequenced, assembled, and analyzed the complete chloroplast genomes of two endemic species from Uzbekistan, *Astragalus nuratensis* and *Oxytropis pseudorosea.* Genome skimming produced high-quality paired-end reads, yielding mean chloroplast sequencing depths of 638.13× for *Astragalus nuratensis* and 1,725.34× for *Oxytropis pseudorosea*, respectively. The chloroplast genomes were 122,316 bp in *A. nuratensis* and 122,708 bp in *O. pseudorosea.* Both genomes encoded 110 unique genes, including 76 protein-coding genes, 30 transfer RNA genes, and 4 ribosomal RNA genes. Consistent with members of the inverted repeat–lacking clade of Fabaceae, both species lacked the typical inverted repeat regions, resulting in a single-copy genome structure. Phylogenetic analysis based on 119 complete chloroplast genomes resolved major lineages within *Astragalus* and related genera with strong support. *Astragalus nuratensis* was placed within the Phaca clade, while *Oxytropis pseudorosea* formed part of a distinct *Oxytropis* lineage. These results provide new genomic resources for understanding evolutionary relationships and plastome evolution in Central Asian legumes.

## Introduction

Chloroplast (cp) genomes of angiosperms have been widely used in studies of phylogeny, sequence variation, genome evolution, and the development of molecular markers ( Dong et al., 2013). In most angiosperms, the cp genome exhibits a conserved quadripartite structure consisting of a large single-copy (LSC) region, a small single-copy (SSC) region, and two inverted repeat (IR) regions ( Sugiura, 1992). Nevertheless, structural modifications, including gene rearrangements, inversions, expansion or loss of IR regions, gene loss, and pseudogene formation, have been documented in several lineages, particularly in Fabaceae ( Cai et al., 2008; Son & Choi, 2022).

Fabaceae is one of the largest families of flowering plants and includes many species of considerable ecological and agricultural importance. According to recent classifications, the family is divided into six subfamilies: Caesalpinioideae, Cercidoideae, Detarioideae, Dialioideae, Duparquetioideae, and Faboideae (Papilionoideae) ( Azani et al., 2017). Within Faboideae, the inverted repeat–lacking clade (IRLC) is characterized by the loss of one copy of the IR region (approximately 25 kb) in the chloroplast genome. This clade comprises about 52 genera and more than 4,000 species, and plastomes of IRLC taxa frequently show gene loss or pseudogenization (e.g., *rps16*, *rpl22*, *infA*, *accD*, and *ycf4*), intron loss ( *clpP*, *atpF*, and *rpoC1*), inversions, and occasional transfer of genes to the nuclear genome ( Son & Choi, 2022).

Within the IRLC, the genera *Astragalus* L. (3,095 species) and *Oxytropis* DC. (608 species) represent highly diverse and taxonomically complex groups ( POWO, 2026). In recent years, complete chloroplast genome sequencing has been increasingly applied to resolve phylogenetic relationships and species boundaries within the genera *Astragalus* and *Oxytropis*. Several chloroplast genomes of species belonging to these genera have been published ( Su et al., 2021; Li et al., 2025; Bobur et al., 2026). Despite these advances, chloroplast genome data remain limited for many narrowly distributed endemic species from Uzbekistan. In the present study, we sequenced, assembled, and analyzed the cp genomes of *Oxytropis pseudorosea* Filim. and *Astragalus nuratensis* Popov, two narrowly distributed endemic species from the Nuratau Mountains of Uzbekistan, Central Asia.

## Methods

Leaf samples of *Oxytropis pseudorosea* and *Astragalus nuratensis* were collected in 2024 from the Nuratau Mountains, Uzbekistan, by Diyorjon Hamrayev. *Astragalus nuratensis* was identified by Beshko Natalya and its voucher specimen was deposited in the National Herbarium of Uzbekistan (TASH) under accession number TASH-2024-AN-001, whereas *Oxytropis pseudorosea* was identified by Doston Turdiyev and its voucher specimen was deposited in TASH under accession number TASH-2024-OP-002.

Genomic DNA was isolated from leaf tissue using the Tiangen DP305 Plant Genomic DNA Kit (Beijing, China). Libraries were prepared with the NEBNext ^®^ Ultra™ DNA Library Prep Kit for Illumina (NEB, USA; Cat. E7370L) following the manufacturer’s protocol, with index codes added during preparation. DNA was sonicated to ~350 bp and fragments were end-repaired, A-tailed, and ligated to Illumina adapters, followed by PCR amplification. PCR products were purified using AMPure XP beads (Beverly, USA). Library quality was assessed on an Agilent 5400 system, and concentrations were quantified by qPCR (1.5 nM). Qualified libraries were pooled and sequenced on Illumina platforms using the PE150 strategy at Novogene (Beijing, China).

Raw reads were assembled *de novo* using NOVOPlasty v4.3.5 ( Dierckxsens et al., 2017), with the plastomes of *Astragalus agrestis* Douglas ex G. Don and *Oxytropis neimonggolica* C.W. Chang & Y.Z. Zhao serving as seed references. The assembly generated a single circular chloroplast genome for each species without structural ambiguities. To assess assembly accuracy and sequencing depth, clean paired-end reads were aligned to the assembled plastomes using BWA-MEM v0.7.17 ( Li, 2013). Alignments were subsequently sorted and indexed with SAMtools v1.19.2 ( Li et al., 2009). Genome annotation was conducted in Geneious v9.0.2 ( Kearse et al., 2012) using closely related reference plastomes. Protein-coding genes, transfer RNAs, and ribosomal RNAs were annotated, and gene boundaries were manually curated to ensure accurate start/stop codons and intron–exon junctions. Furthermore, the cp map was generated using Chloroplot ( https://irscope.shinyapps.io/chloroplot/) ( Zheng et al., 2020).

For several taxa included in the phylogenetic analysis, complete chloroplast genome sequences were not available in GenBank. Only raw sequencing data (SRA accessions; SRR numbers listed in Table 1) were available. Therefore, the chloroplast genomes of these taxa were assembled *de novo* using NOVOPlasty v4.3.5 ( Dierckxsens et al., 2017). A total of 119 complete chloroplast genome sequences were included in the phylogenetic analysis. Of these, two plastomes were newly sequenced in this study, while the remaining 117 sequences were downloaded from the NCBI GenBank database ( Table 1 **)**. Complete chloroplast genome sequences were aligned using MAFFT ( Katoh & Standley, 2013). The alignment was manually inspected and used to reconstruct phylogenetic relationships under the maximum likelihood criterion in IQ-TREE 2 ( Minh et al., 2020). The best-fit substitution model was selected using ModelFinder ( Kalyaanamoorthy et al., 2017), and branch support was assessed with 1,000 ultrafast bootstrap replicates ( Hoang et al., 2018).

**Table 1.** List of taxa used in the phylogenetic analysis, including their respective GenBank accession numbers. For taxa lacking published chloroplast genome accessions, plastomes were assembled in this study from raw reads downloaded from the NCBI Sequence Read Archive (SRR accessions provided).

Species	Accession	Species	Accession
*Astragalus camptodontus*	SRR12136586	*A. aksuensis*	SRR12136588
*A. confertus*	SRR12136589	*A. massanderanus*	SRR12136591
*A. handelii*	SRR12136594	*A. zacharensis*	SRR12136595
*A. uliginosus*	SRR12136597	*A. koburensis*	SRR12136600
*A. beketowii*	SRR12136611	*A. jessenii*	SRR12136613
*A. tibetanus*	SRR12136615	*A. dendroides*	SRR12136619
*A. candissimus*	SRR12136622	*A. platyphyllus*	SRR12136623
*A. himalayanus*	SRR12136624	*A. roseus*	SRR12136625
*A. stalinskii*	SRR12136626	*A. orbicularifolius*	SRR12136627
*A. hamosus*	SRR12136635	*A. heinsensis*	SRR12136637
*A. hoantchy*	SRR12136647	*A. saccolacys*	SRR12136648
*A. neomondelphus*	SRR12136653	*A. schmalhausenii*	SRR12136654
*A. zingeri*	SRR12136656	*A. eximius*	SRR12136657
*A. xanthomeloides*	SRR12136658	*A. piletocladus*	SRR12136659
*A. parrowianus*	SRR12136660	*A. coluteocarpus*	SRR12136661
*A. thurberi*	SRR12136664	*A. americanus*	SRR12136665
*A. peduncularis*	SRR12136666	*A. dipelta*	SRR12136669
*A. acaulis*	PV802397	*A. austrotristis*	NC_063479
*A. adsurgens*	NC_085710	*A. angustidens*	ON550413
*A. agrestis*	PP328800	*A. alpinus*	NC_063482
*A. ampullarius*	PV870668	*A. arbuscula*	ON550410
*A. arpilobus*	NC_063483	*A. arrectus*	NC_047381
*A. bhotanensis*	NC_063484	*A. biristatus*	MZ923743
*A. calycosus var. calycosus*	MZ725323	*A. canadensis var. brevidens*	PV870770
*A. chinensis*	NC_063486	*A. clevelandii*	ON550409
*A. cognatus*	MZ127832	*A. chinense*	ON550407
*A. dilutus*	PV870843	*A. effusus*	MZ901207
*A. flexuosus var. flexuosus*	ON550403	*A. flexus*	NC_083391
*A. floridus*	NC_058825	*A. galactites*	OY754317
*A. glycyphyllos*	MY746310	*A. gummifer*	NC_063487
*A. gypsodes*	LC764834	*A. iranicus*	NC_077545
*A. juanquensis*	PV798172	*A. khasianus*	ON550401
*A. laksamanni*	NC_085734	*A. leucocephalus*	ON550399
*A. lithophilus*	PV870772	*A. lonchocarpus*	LC764835
*A. macropelmatus*	OP723863	*A. melilotoides* var. *tenuis*	OP723862
*A. membranaceus*	PV761671	*A. membranaceus* var. *membranaceus*	KX255662
*A. moseleios*	LC764836	*A. mongholicus*	OR712437
*A. mongholicus* var. *dahuricus*	MW719856	*A. mongholicus* var. *nakaianus*	KR296789
*A. muliensis*	NC_083909	*A. neglectus*	NC_063488
*A. nuratensis*	PX928918	*A. obscurus*	NC_063489
*A. odoratus*	LC764837	*A. oxylottis*	NC_077543
*A. pallasi*	NC_077542	*A. polysladus*	PV910879
*A. psilosepalus*	NC_083910	*A. purpurinus*	OR652287
*A. rumpens*	PX711949	*A. scaberrimus*	MW654102
*A. sereno* var. *sereno*	MZ923754	*A. sieversianus*	NC_077540
*A. sinicus*	OM287552	*A. stipulatus*	OR491698
*A. strictus*	MT120746	*A. toanus var. toanus*	MZ923756
*A. tongolensis*	PX754582	*A. tumabtsica*	NC_083912
*A. vulpinus*	ON550388	*A. wootonii* var. *wootonii*	MZ923757
*A. yunnanensis* subsp. *incanus*	PV156653	*A. zerabulaki*	PX711948
*Oxytropis chiliophylla*	PV694277	*O. filiformis*	PV684033
*O. latibracteata*	PV694278	*Oplonia microphylla*	PV684029
*O. neimongolica*	PV684030	*O. proboscidea*	PV694280
*O. pseudorosea*	PX512474	*O.* sp. TT4869–1	PX652196
*Tibetia liangshanensis*	MF193597	*Alhagi sparsifolia*	MW349013
*Caragana korshinskii*	KX289923	*Hedysarum taipeicum*	MK426698
*Caragana microphylla*	KX289922	*Corethrodendron multijugum*	OP748243
*Carmichaelia australis*	PV870797	*Phyllolobium chinense*	MZ221114
*Phyllolobium camptodontum*	SRR12136586	*Lessertia frutescens*	MF286764
*Sphaerophysa salsula*	PV870675		

## Results

Genome skimming generated a total of 39,668,027 paired-end reads for *A. nuratensis* and 16,998,801 paired-end reads for *O. pseudorosea.* After quality filtering, 39,666,696 and 16,994,332 reads were retained as high-quality reads for *A. nuratensis* and *O. pseudorosea*, respectively. Mapping of the filtered reads to the assembled chloroplast reference genomes showed that 540,829 reads (1.36%) in *A. nuratensis* and 1,421,542 reads (8.36%) in *O. pseudorosea* were successfully aligned. Properly paired reads accounted for 1.33% and 8.27% of the total reads, respectively. The chloroplast genome of *Astragalus nuratensis* was recovered with an average sequencing depth of 638.13×, with coverage ranging from 9× to 3273×. In comparison, the chloroplast genome of *Oxytropis pseudorosea* exhibited a substantially higher mean sequencing depth of 1,725.34×, with coverage ranging from 12× to 2146× ( Figure 1).

**Figure 1.**
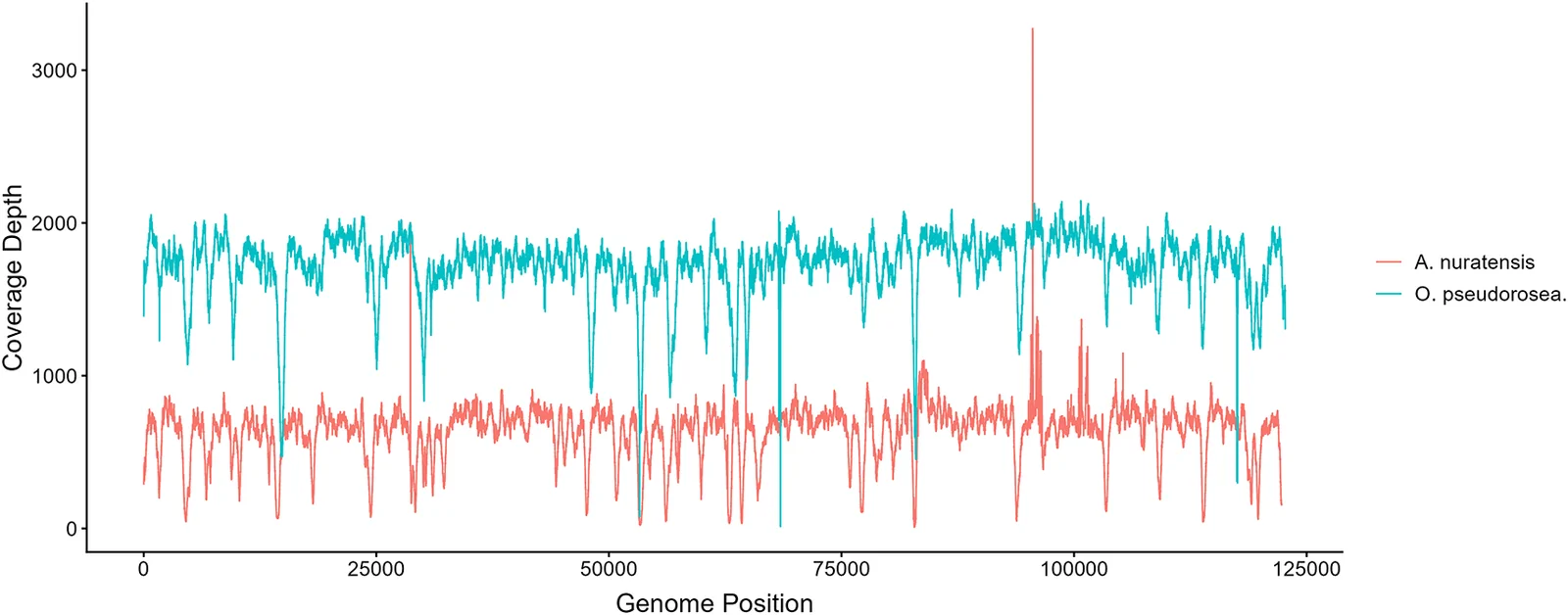
Coverage depth across the chloroplast genomes of *Astragalus nuratensis* and *Oxytropis pseudorosea*.

The complete chloroplast genome of *O. pseudorosea* was 122,708 bp in length, whereas that of *A. nuratensis* was 122,316 bp. Both genomes encoded a total of 110 unique genes, including 77 protein-coding genes (CDS), 30 transfer RNA (tRNA) genes, and 4 ribosomal RNA (rRNA) genes ( Figure 2). In both *Astragalus nuratensis* and *Oxytropis pseudorosea*, the genes *rpl16, rpl2, rpoC1, ndhA, ndhB, petB, petD, atpF, clpP, trnA-UGC, trnG-UCC, trnI-GAU, trnK-UUU, trnL-UAA, and trnV-UAC* each contain one intron, while *ycf3* contains two introns. In addition *, rps12* is a trans-spliced gene with two introns (one cis-spliced and one trans-spliced). Consistent with other members of the inverted repeat–lacking clade (IRLC) of legumes, the typical inverted repeat regions were absent in both species, resulting in a single-copy chloroplast genome structure, a feature commonly reported in plastome studies of *Astragalus* and related taxa ( Moghaddam et al., 2023; Ma et al., 2025; Li et al., 2025).

**Figure 2.**
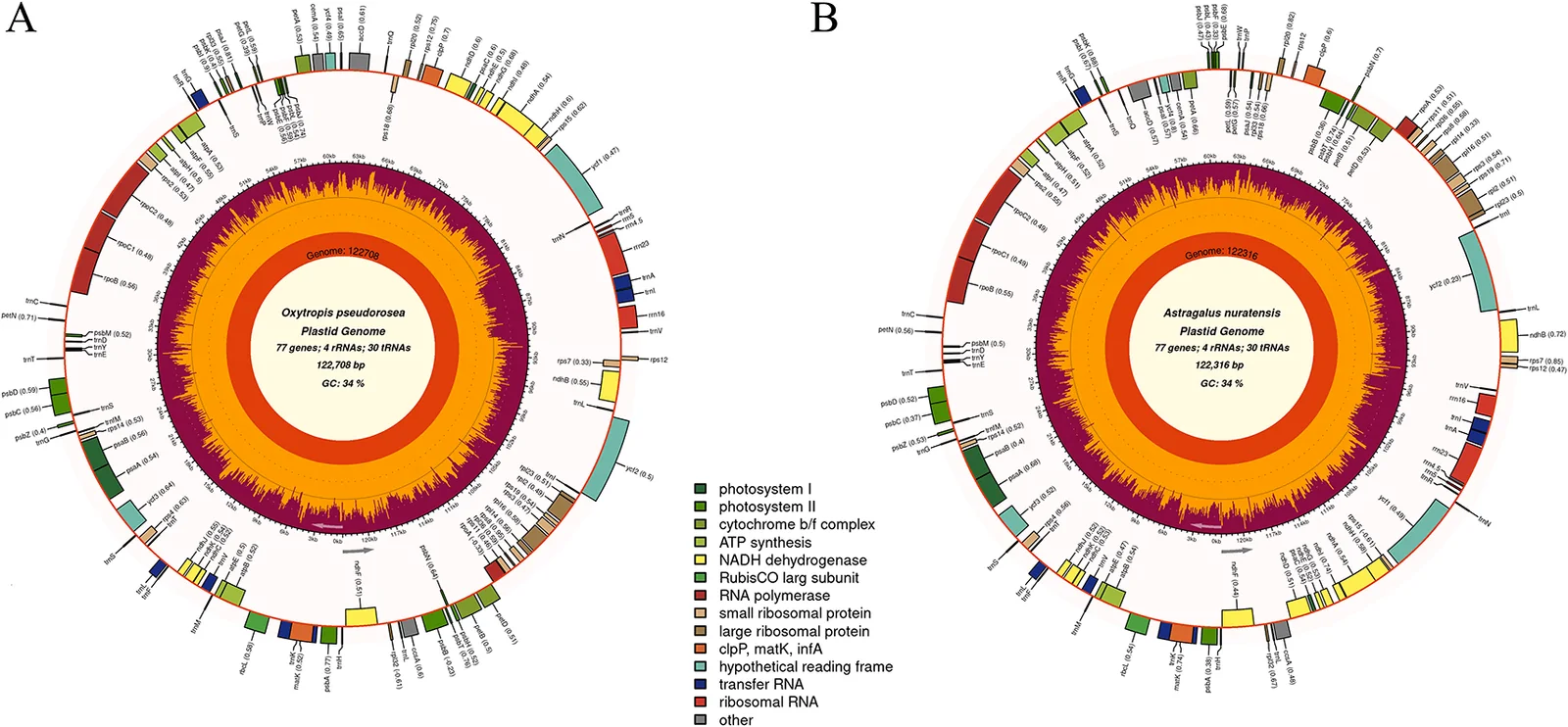
The circular maps of the chloroplast genomes of *Oxytropis pseudorosea* and *Astragalus nuratensis* were drawn using Chloroplot. The innermost circle represents the lengths of chloroplast genomes. The second concentric ring illustrates nucleotide composition, where the orange segment indicates GC content and the yellow segment corresponds to AT content. The outermost circle displays the distribution of genes, which are color-coded according to their functional classification. The transcriptional orientation is indicated such that genes on the inner circle are transcribed clockwise, whereas those on the outer circle are transcribed anticlockwise.

Maximum likelihood analysis based on complete chloroplast genome sequences resolved the sampled taxa into several well-supported clades corresponding to recognized infrageneric groups within *Astragalus* and related genera ( Figure 3). Most backbone nodes received strong bootstrap support (BS ≥ 95), indicating a stable phylogenetic structure. The overall topology was consistent with previous phylogenomic studies of *Astragalus*, which also recovered major lineages with strong support using plastome and target-enrichment data ( Su et al., 2021; Buono et al., 2025).

**Figure 3.**
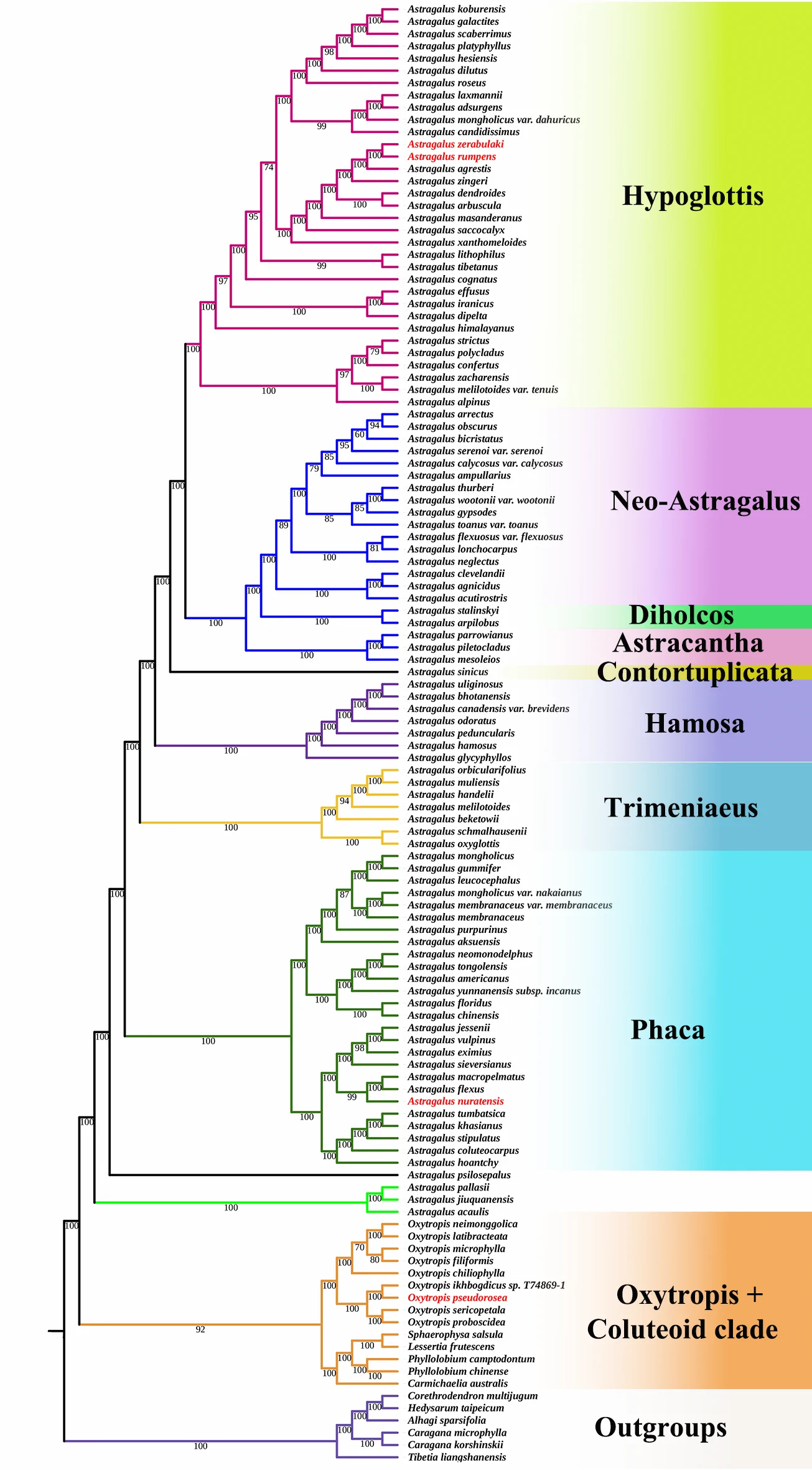
Maximum likelihood phylogenetic tree inferred from complete chloroplast genome sequences showing the relationships of *Astragalus nuratensis*, *Oxytropis pseudorosea*, and other members of the inverted repeat–lacking clade (IRLC).

Species of *Astragalus* were distributed among the major lineages corresponding to the Hypoglottis, Neo-Astragalus, Diholcos, Astracantha, Contortuplicata, Hamosa, Trimeniaeus, and Phaca clades. Among these, the Phaca clade represented one of the largest lineages and included *A. nuratensis*, which grouped with other members of this clade with strong bootstrap support. Species of *Oxytropis*, including *O. pseudorosea*, formed a distinct and well-supported lineage corresponding to the *Oxytropis* + Coluteoid clade, clearly separated from the main *Astragalus* lineages, in agreement with previous plastome-based phylogenies ( Buono et al., 2025).

In the phylogenetic tree, *A. zerabulaki*, another endemic species, was resolved within the Hypoglottis clade and formed a well-supported sister relationship with *A. rumpens*, indicating close evolutionary relationships among members of this lineage.

## Data Availability

NCBI BioProject: Raw sequencing data for *Astragalus nuratensis* and *Oxytropis pseudorosea.* Accession number PRJNA1425239. https://www.ncbi.nlm.nih.gov/bioproject/PRJNA1425239 ( Karimov, 2026a). NCBI Sequence Read Archive (SRA): Raw sequencing data of *Astragalus nuratensis* and *Oxytropis pseudorosea.* Accession numbers SRR37271903 and SRR37271902; https://www.ncbi.nlm.nih.gov/sra/?term=SRR37271903; https://www.ncbi.nlm.nih.gov/sra/?term=SRR37271902 ( Karimov, 2026b). NCBI GenBank: Chloroplast genomes of *Astragalus nuratensis* and *Oxytropis pseudorosea.* Accession numbers PX928918 and PX512474; https://www.ncbi.nlm.nih.gov/nuccore/PX928918.1; https://www.ncbi.nlm.nih.gov/nuccore/PX512474.1 ( Karimov, 2026c). Data are available under the terms of the Creative Commons Attribution 4.0 International license (CC-BY 4.0).
