# Supplementary material for: Complete chloroplast genomes of endemic Astragalus and Oxytropis species from Uzbekistan
Source: F1000Res. 2026 Jul 14;15:445. Originally published 2026 Mar 27. [Version 4] doi: 10.12688/f1000research.178398.4 (PMC13555028; doi:10.12688/f1000research.178398.4)
Supplement: Peer review history

# Reviewer report: Reviewer response for version 4

**DOI:** 10.5256/f1000research.203901.r501462

**Published:** 2026-07-15

**Authors:** Hoang Dang Khoa Do

**Affiliations:** 1 Nguyen Tat Thanh University, Ho Chi Minh City, Ho Chi Minh, Vietnam

The authors revised the manuscript based on the reviewer's comments.

 The revised manuscript is now suitable for indexing.

Are the datasets clearly presented in a usable and accessible format, and the assembly and annotation available in an appropriate subject-specific repository?

Yes

Are sufficient details of the sequencing and extraction, software used, and materials provided to allow replication by others?

Partly

Are the rationale for sequencing the genome and the species significance clearly described?

Yes

Are the protocols appropriate and is the work technically sound?

Yes

Reviewer Expertise:

Plant Chloroplast genomes

I confirm that I have read this submission and believe that I have an appropriate level of expertise to confirm that it is of an acceptable scientific standard.

# Reviewer report: Reviewer response for version 3

**DOI:** 10.5256/f1000research.202456.r489947

**Published:** 2026-06-03

**Authors:** Hoang Dang Khoa Do

**Affiliations:** 1 Nguyen Tat Thanh University, Ho Chi Minh City, Ho Chi Minh, Vietnam

Dear Authors,

 In the Introduction section, there are two sentences.

 "This clade comprises about 52 genera and more than 4,000 species, and plastomes of IRLC taxa frequently show gene loss or pseudogenization (e.g., rps16, rpl22, infA, accD, and ycf4), intron loss (clpP, atpF, and rpoC1), inversions, and occasional transfer of genes to the nuclear genome (Son & Choi, 2022)."

 "Within the IRLC, the genera Astragalus L. (3,095 species) and Oxytropis DC. (608 species) represent highly diverse and taxonomically complex groups (POWO, 2026)."

 I am not sure the reason why the gene names and genus names were not italic in the above two sentences although the authors responsed that they fixed it.

 Please check and revise.

 Thank you.

Are the datasets clearly presented in a usable and accessible format, and the assembly and annotation available in an appropriate subject-specific repository?

Yes

Are sufficient details of the sequencing and extraction, software used, and materials provided to allow replication by others?

Partly

Are the rationale for sequencing the genome and the species significance clearly described?

Yes

Are the protocols appropriate and is the work technically sound?

Yes

Reviewer Expertise:

Plant Chloroplast genomes

I confirm that I have read this submission and believe that I have an appropriate level of expertise to confirm that it is of an acceptable scientific standard, however I have significant reservations, as outlined above.

# Response

**Published:** 2026-06-04

**Authors:** Bobur Karimov

**Affiliations:** Institute of Botany of the Academy of Sciences of the Republic of Uzbekistan, Tashkent, Tashkent Province, Uzbekistan

Thank you for your comment.

 This issue was corrected in the revised manuscript submitted by the authors. The genus names and the gene names cited in these sentences were formatted in italics. The current proof appears to have lost this formatting during copy editing or typesetting.

 We kindly ask the production team to restore the appropriate italic formatting

 Thank you.

# Response

**Published:** 2026-06-12

**Authors:** Bobur Karimov

**Affiliations:** Institute of Botany of the Academy of Sciences of the Republic of Uzbekistan, Tashkent, Tashkent Province, Uzbekistan

The formatting has now been carefully corrected throughout the manuscript, including the two sentences highlighted.

# Reviewer report: Reviewer response for version 2

**DOI:** 10.5256/f1000research.200633.r484854

**Published:** 2026-05-22

**Authors:** Hoang Dang Khoa Do

**Affiliations:** 1 Nguyen Tat Thanh University, Ho Chi Minh City, Ho Chi Minh, Vietnam

Dear Authors, 

 Thank you very much for revising the manuscript based on the reviewer's comments.

 Most of my concerns were resolved.

 However, there is still a minor issue about the italic font for gene names (rps16, rpl22, infA, accD, and ycf4; clpP, atpF, and rpoC1) and genus name (Oxytropis) in the Introduction.

 Thank you.

Are the datasets clearly presented in a usable and accessible format, and the assembly and annotation available in an appropriate subject-specific repository?

Yes

Are sufficient details of the sequencing and extraction, software used, and materials provided to allow replication by others?

Partly

Are the rationale for sequencing the genome and the species significance clearly described?

Yes

Are the protocols appropriate and is the work technically sound?

Yes

Reviewer Expertise:

Plant Chloroplast genomes

I confirm that I have read this submission and believe that I have an appropriate level of expertise to confirm that it is of an acceptable scientific standard, however I have significant reservations, as outlined above.

# Response

**Published:** 2026-05-25

**Authors:** Bobur Karimov

**Affiliations:** Institute of Botany of the Academy of Sciences of the Republic of Uzbekistan, Tashkent, Tashkent Province, Uzbekistan

Dear Reviewer,

 Thank you very much for your careful review and for your positive comments on our revised manuscript.

 We appreciate your additional observation regarding the formatting issue. We have carefully checked the manuscript and corrected the italic formatting for all gene names ( *rps16, rpl22, infA, accD, ycf4, clpP, atpF,* and *rpoC1*) as well as the genus name *Oxytropis* in the Introduction.

 These corrections have been incorporated into the revised manuscript.

 Thank you again for your valuable comments and suggestions.

 Sincerely,

 The Authors

# Reviewer report: Reviewer response for version 2

**DOI:** 10.5256/f1000research.200633.r484855

**Published:** 2026-05-20

**Authors:** Yoshinori Fukasawa

**Affiliations:** 1 Center for Bioscience Research and Education Utsunomiya University, Tochigi, Japan

Dear Authors,

 Thank you for addressing the comments. The revised manuscript has been substantially improved. I am satisfied that the concerns raised in my initial review have been adequately addressed and am happy to approve this article.

Are the datasets clearly presented in a usable and accessible format, and the assembly and annotation available in an appropriate subject-specific repository?

Yes

Are sufficient details of the sequencing and extraction, software used, and materials provided to allow replication by others?

Partly

Are the rationale for sequencing the genome and the species significance clearly described?

Yes

Are the protocols appropriate and is the work technically sound?

Yes

Reviewer Expertise:

Plant genomics and bioinformatics

I confirm that I have read this submission and believe that I have an appropriate level of expertise to confirm that it is of an acceptable scientific standard.

# Reviewer report: Reviewer response for version 1

**DOI:** 10.5256/f1000research.196779.r473037

**Published:** 2026-04-27

**Authors:** Yoshinori Fukasawa

**Affiliations:** 1 Center for Bioscience Research and Education Utsunomiya University, Tochigi, Japan

Dear Authors,

 Thank you for reporting the complete chloroplast genomes of *Astragalus nuratensis* and *Oxytropis pseudorosea*. The addition of new plastome resources for Astragalus and Oxytropis—two of the largest and most taxonomically challenging genera in the inverted repeat–lacking clade (IRLC)—is a welcome contribution, particularly given the ongoing interest in plastome evolution within this clade. Below I offer several suggestions that I believe would strengthen the manuscript.

 1. Citation for the conserved quadripartite structure of chloroplast genomes

 In the Introduction, the conserved quadripartite structure of angiosperm chloroplast genomes is cited as (Karimov et al. 2025). However, this is a well-established and broadly recognized feature of plastome architecture that has been described in numerous foundational works. The cited reference reports chloroplast genomes of Cousinia (Asteraceae) species and, while a valuable study in its own right, does not serve as a primary or representative source for this general statement. I would suggest replacing it with one or more widely cited references that originally established this structural model.

 2. Chloroplast genome map of *Oxytropis pseudorosea*

 Figure 1 presents the chloroplast genome map of *A. nuratensis* only. Since the manuscript reports two newly sequenced plastomes, I would recommend including a corresponding genome map of *O. pseudorosea* as well, either as an additional panel in Figure 1 or as a separate figure. This would provide a more balanced presentation of the two genomes.

 3. Discrepancy in gene counts between Figure 1 and the text

 The main text states that both genomes encode 76 protein-coding genes, 30 tRNA genes, and 4 rRNA genes (110 unique genes total). However, the annotation within Figure 1 indicates "77 genes; 4 rRNAs; 30 tRNAs" for *A. nuratensis*. Please verify and reconcile these numbers to ensure consistency between the figure and the text.

 4. Assembly validation metrics

 The authors report mapping clean reads back to the assembled plastomes using BWA-MEM, which is an appropriate approach. However, the current manuscript reports only the mean sequencing depth. To further support the completeness and accuracy of the assemblies, I would suggest briefly reporting additional validation metrics, such as: (i) whether any gaps or regions of zero coverage were observed, (ii) the minimum and maximum coverage across the genome, and/or (iii) a coverage depth plot provided as supplementary material. These additions would allow readers to better assess the quality of the assembled sequences.

 5. Reporting gene loss, pseudogenization, and intron loss in the IRLC context

 The Introduction appropriately highlights that IRLC plastomes frequently exhibit gene loss or pseudogenization (e.g., *rps16, rpl22, infA, accD, ycf4*) and intron loss (e.g., in *clpP, atpF, rpoC1*). However, the Results section does not describe which of these events, if any, were observed in the two newly sequenced plastomes. Even a brief statement confirming or contrasting the gene/intron content of *A. nuratensis* and *O. pseudorosea *with that of other IRLC members would add informative context to the genome descriptions and enhance the value of this report as a genomic resource.

 Summary

 This manuscript provides new plastome data for two IRLC species from Central Asia and meets the essential criteria for a Genome Note. The assembly and analytical approaches are technically appropriate.

Are the datasets clearly presented in a usable and accessible format, and the assembly and annotation available in an appropriate subject-specific repository?

Yes

Are sufficient details of the sequencing and extraction, software used, and materials provided to allow replication by others?

Partly

Are the rationale for sequencing the genome and the species significance clearly described?

Yes

Are the protocols appropriate and is the work technically sound?

Yes

Reviewer Expertise:

Plant genomics and bioinformatics

I confirm that I have read this submission and believe that I have an appropriate level of expertise to confirm that it is of an acceptable scientific standard, however I have significant reservations, as outlined above.

# Response

**Published:** 2026-04-29

**Authors:** Bobur Karimov

**Affiliations:** Institute of Botany of the Academy of Sciences of the Republic of Uzbekistan, Tashkent, Tashkent Province, Uzbekistan

**Reviewer 2**

 We sincerely thank the reviewer for the careful evaluation of our manuscript and for the constructive comments, which have helped improve the clarity and quality of the study. Below we provide point-by-point responses.

**1. Citation for the conserved quadripartite structure of chloroplast genomes**

 In the Introduction, the conserved quadripartite structure of angiosperm chloroplast genomes is cited as (Karimov et al. 2025). However, this is a well-established and broadly recognized feature of plastome architecture that has been described in numerous foundational works. The cited reference reports chloroplast genomes of Cousinia (Asteraceae) species and, while a valuable study in its own right, does not serve as a primary or representative source for this general statement. I would suggest replacing it with one or more widely cited references that originally established this structural model.

**Response:** A more appropriate and widely accepted reference has been added to support the conserved quadripartite structure of angiosperm chloroplast genomes.

**2. Chloroplast genome map of Oxytropis pseudorosea**

 Figure 1 presents the chloroplast genome map of A. nuratensis only. Since the manuscript reports two newly sequenced plastomes, I would recommend including a corresponding genome map of O. pseudorosea as well, either as an additional panel in Figure 1 or as a separate figure. This would provide a more balanced presentation of the two genomes.

**Response:** The chloroplast genome map of *Oxytropis pseudorosea* has been added accordingly to provide balanced visualization of both plastomes.

**3. Discrepancy in gene counts between Figure 1 and the text**

 The main text states that both genomes encode 76 protein-coding genes, 30 tRNA genes, and 4 rRNA genes (110 unique genes total). However, the annotation within Figure 1 indicates "77 genes; 4 rRNAs; 30 tRNAs" for A. nuratensis. Please verify and reconcile these numbers to ensure consistency between the figure and the text.

**Response:** The gene annotation has been carefully checked, and inconsistencies between the figure and the text have been corrected to ensure full consistency.

**4. Assembly validation metrics**

 The authors report mapping clean reads back to the assembled plastomes using BWA-MEM, which is an appropriate approach. However, the current manuscript reports only the mean sequencing depth. To further support the completeness and accuracy of the assemblies, I would suggest briefly reporting additional validation metrics, such as: (i) whether any gaps or regions of zero coverage were observed, (ii) the minimum and maximum coverage across the genome, and/or (iii) a coverage depth plot provided as supplementary material. These additions would allow readers to better assess the quality of the assembled sequences.

**Response:** Additional assembly validation metrics have been included, including minimum and maximum coverage values, assessment of zero-coverage regions, and a coverage depth plot provided as Figure 1.

**5. Reporting gene loss, pseudogenization, and intron loss in the IRLC context**

 The Introduction appropriately highlights that IRLC plastomes frequently exhibit gene loss or pseudogenization (e.g., rps16, rpl22, infA, accD, ycf4) and intron loss (e.g., in clpP, atpF, rpoC1). However, the Results section does not describe which of these events, if any, were observed in the two newly sequenced plastomes. Even a brief statement confirming or contrasting the gene/intron content of A. nuratensis and O. pseudorosea with that of other IRLC members would add informative context to the genome descriptions and enhance the value of this report as a genomic resource.

**Response:** A brief description of gene content and intron structure has been added to the Results comparison with typical IRLC characteristics.

# Reviewer report: Reviewer response for version 1

**DOI:** 10.5256/f1000research.196779.r471852

**Published:** 2026-04-03

**Authors:** Hoang Dang Khoa Do

**Affiliations:** 1 Nguyen Tat Thanh University, Ho Chi Minh City, Ho Chi Minh, Vietnam

Dear Authors,

 Thank you very much for adding new genomic data about Astragalus and Oxytropis species. The content of the manuscript is suitable for Genome Note of F1000 Research.

 Please check my comment below.

 1/ Please update the role of authors because most authors are for writing the manuscript. Who did the sampling, experiment, and analyzing the data?

 2/ "Chloroplast (cp) genomes of angiosperms have been widely used in studies of phylogeny, sequence variation, genome evolution, and the development of molecular markers." Please add references for this statement.

 3/ "In most angiosperms, the cp genome exhibits a conserved quadripartite structure consisting of a large single-copy (LSC) region, a small single-copy (SSC) region, and two inverted repeat (IR) regions (Karimov et al., 2025)." the current reference is not suitable for the statement because Karimov et al 2025 only reported complete chloroplast genomes of six Cousinia (Asteraceae) species which are not representative for most angiosperms.

 4/ Please use italic font for gene names in the Introduction part.

 5/ It would be better if the authors add introduction about the published chloroplast genomes of Astragalus and Oxytropis, especially Astragalus zerabulaki which was mentioned at the end of the manuscript.

 6/ "Voucher specimens were deposited in the National Herbarium of Uzbekistan (TASH) under the accession numbers TASH-2024-AN-001 and TASH-2024-OP-002."; "Genome skimming generated high-quality paired-end reads, enabling the recovery of complete plastomes with mean sequencing depths of 638× and 1,725×, respectively." please revise these sentences to clarify which feature is of Astragalus nuratensis and which information belongs to Oxytropis pseudorosea.

 7/ "High-quality reads were assembled de novo using NOVOPlasty v4.3.5" how to get high-quality reads? Please add.

 8/ "For several taxa included in the phylogenetic analysis, complete chloroplast genome sequences were not available in GenBank. Only raw sequencing data (SRA accessions; SRR numbers listed in Table 1) were available. Therefore, the chloroplast genomes of these taxa were assembled." How to complete chloroplast genomes from SRA data? Please add.

 9/ Because the manuscript reports two chloroplast genome, it would be better if the authors add the chloroplast genome map of O. pseudorosea in Figure 1.

 10/ "Table 1. List of taxa included in the phylogenetic analysis, accession numbers." Please revise Table 1 caption.

 11/ Please correct accession number of O. pseudorosea which is not PV684035 from GenBank database in Table 1.

 Thank you.

Are the datasets clearly presented in a usable and accessible format, and the assembly and annotation available in an appropriate subject-specific repository?

Yes

Are sufficient details of the sequencing and extraction, software used, and materials provided to allow replication by others?

Partly

Are the rationale for sequencing the genome and the species significance clearly described?

Yes

Are the protocols appropriate and is the work technically sound?

Yes

Reviewer Expertise:

Plant Chloroplast genomes

I confirm that I have read this submission and believe that I have an appropriate level of expertise to confirm that it is of an acceptable scientific standard, however I have significant reservations, as outlined above.

# Response

**Published:** 2026-04-29

**Authors:** Bobur Karimov

**Affiliations:** Institute of Botany of the Academy of Sciences of the Republic of Uzbekistan, Tashkent, Tashkent Province, Uzbekistan

**Reviewer 1**

 We sincerely thank the reviewer for the careful evaluation of our manuscript and for the valuable comments, which helped improve the quality of the work. Below we provide point-by-point responses.

**Comment 1/** Please update the role of authors because most authors are for writing the manuscript. Who did the sampling, experiment, and analyzing the data?

**Response:** The system did not provide an option to edit and resubmit the updated version with revised author contributions.

**Comment 2/** "Chloroplast (cp) genomes of angiosperms have been widely used in studies of phylogeny, sequence variation, genome evolution, and the development of molecular markers." Please add references for this statement.

**Response:** Appropriate references have been added to support this statement in the revised manuscript.

**Comment 3/** "In most angiosperms, the cp genome exhibits a conserved quadripartite structure consisting of a large single-copy (LSC) region, a small single-copy (SSC) region, and two inverted repeat (IR) regions (Karimov et al., 2025)." the current reference is not suitable for the statement because Karimov et al 2025 only reported complete chloroplast genomes of six Cousinia (Asteraceae) species which are not representative for most angiosperms.

**Response:** A more appropriate and widely accepted reference has been added to support this statement.

**Comment 4/** Please use italic font for gene names in the Introduction part.

**Response:** Gene names in the Introduction have been corrected and formatted in italics.

**Comment 5/** It would be better if the authors add introduction about the published chloroplast genomes of Astragalus and Oxytropis, especially Astragalus zerabulaki which was mentioned at the end of the manuscript.

**Response:** A new introductory paragraph has been added covering published chloroplast genomes of *Astragalus* and *Oxytropis*

**Comment 6/** "Voucher specimens were deposited in the National Herbarium of Uzbekistan (TASH) under the accession numbers TASH-2024-AN-001 and TASH-2024-OP-002."; "Genome skimming generated high-quality paired-end reads, enabling the recovery of complete plastomes with mean sequencing depths of 638× and 1,725×, respectively." please revise these sentences to clarify which feature is of Astragalus nuratensis and which information belongs to Oxytropis pseudorosea.

**Response:** The sentences have been revised to clearly distinguish species-specific voucher information and sequencing depth values.

**Comment 7/** "High-quality reads were assembled de novo using NOVOPlasty v4.3.5" how to get high-quality reads? Please add.

**Response:** Raw sequencing reads were directly used, and NOVOPlasty performs internal quality filtering during the assembly process.

**Comment 8/** "For several taxa included in the phylogenetic analysis, complete chloroplast genome sequences were not available in GenBank. Only raw sequencing data (SRA accessions; SRR numbers listed in Table 1) were available. Therefore, the chloroplast genomes of these taxa were assembled." How to complete chloroplast genomes from SRA data? Please add.from SRA to novoplasty

**Response:** Raw sequencing data were downloaded from SRA and assembled into chloroplast genomes using NOVOPlasty following the same assembly workflow.

**Comment 9/** Because the manuscript reports two chloroplast genome, it would be better if the authors add the chloroplast genome map of O. pseudorosea in Figure 1.

**Response:** The chloroplast genome map of *Oxytropis pseudorosea* has been added to Figure 1.

**Comment 10/** "Table 1. List of taxa included in the phylogenetic analysis, accession numbers." Please revise Table 1 caption.

**Response:** The caption of Table 1 has been revised for clarity and consistency.

**Comment 11/** Please correct accession number of O. pseudorosea which is not PV684035 from GenBank database in Table 1.

**Response:** The accession number has been corrected according to the GenBank database.
